# Cardiolipin, the Mitochondrial Signature Lipid: Implication in Cancer

**DOI:** 10.3390/ijms21218031

**Published:** 2020-10-28

**Authors:** Seyedeh Tayebeh Ahmadpour, Karine Mahéo, Stéphane Servais, Lucie Brisson, Jean-François Dumas

**Affiliations:** Université de Tours, Inserm, Nutrition, Croissance et Cancer UMR1069, 37032 Tours, France; seyedehtayebeh.ahmadpour@etu.univ-tours.fr (S.T.A.); karine.maheo@univ-tours.fr (K.M.); stephane.servais@univ-tours.fr (S.S.); lucie.brisson@univ-tours.fr (L.B.)

**Keywords:** cardiolipin, cancer, mitochondria

## Abstract

Cardiolipins (CLs) are specific phospholipids of the mitochondria composing about 20% of the inner mitochondria membrane (IMM) phospholipid mass. Dysregulation of CL metabolism has been observed in several types of cancer. In most cases, the evidence for a role for CL in cancer is merely correlative, suggestive, ambiguous, and cancer-type dependent. In addition, CLs could play a pivotal role in several mitochondrial functions/parameters such as bioenergetics, dynamics, mitophagy, and apoptosis, which are involved in key steps of cancer aggressiveness (i.e., migration/invasion and resistance to treatment). Therefore, this review focuses on studies suggesting that changes in CL content and/or composition, as well as CL metabolism enzyme levels, may be linked with the progression and the aggressiveness of some types of cancer. Finally, we also introduce the main mitochondrial function in which CL could play a pivotal role with a special focus on its implication in cancer development and therapy.

## 1. Introduction 

It has long been thought that cancer cells do not produce energy by mitochondrial oxidative phosphorylation but through a high glycolytic rate coupled with lactic acid production even in presence of oxygen namely the Warburg effect (or aerobic glycolysis) [1]. This reprogramming of metabolism that favors macromolecule biosynthesis, affects tumor microenvironment, and confers direct signaling functions to tumor cells resulting in tumor growth has been documented for over 90 years. However, it is still debated [2]. Contrary to the initial proposition [1], it is now recognized that mitochondrial oxidative phosphorylation (OXPHOS) is not dysfunctional in all cancer cells and participates in ATP production [3]. More generally, through its important contribution to the production of ATP, macromolecules, apoptosis, and oxidative stress, it is now well accepted that mitochondria are involved in key steps of cancer aggressiveness, sustaining tumor growth and cancer progression [2,4,5].

**Figure 1:**
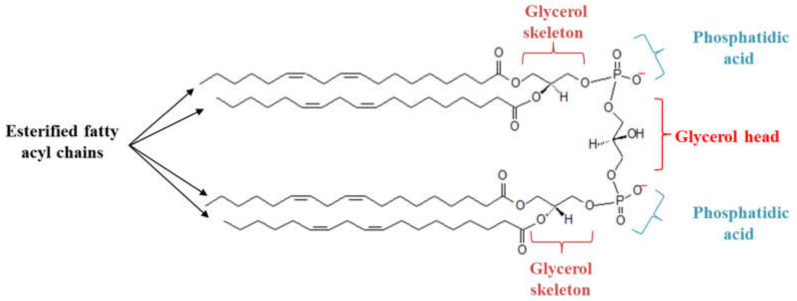
Structure of cardiolipin (CL). Each CL possesses a glycerol head group bound to two phosphatidyl moieties, forming an anionic polar head group. The presence of four esterified fatty acyl chains bound to the glycerol head group forms a cone-shaped structure.

Mitochondrial membranes are made up of different phospholipids: Phosphatidylcholine, phosphatidylethanolamine, phosphatidylinositol, phosphatidylserine, phosphatidic acid, and cardiolipins [6]. Cardiolipins (CLs) are specific phospholipids of the mitochondria comprising about 20% of the inner mitochondria membrane (IMM) phospholipids mass [6]. Each CL possesses a glycerol head group bound to two phosphatidyl moieties, forming an anionic polar head group. The presence of four esterified fatty acyl chains bound to the glycerol head group forms a cone-shaped structure [7] (Figure 1). CL molecular species differ across organisms and tissues and a specific remodeling of CL’s acyl chain is observed in several pathologies. CLs interact with IMM proteins and enzymes that are involved in the mitochondrial electron transfer chain (ETC) and the oxidative phosphorylation system (OXPHOS), ensuring the mitochondrial cristae organization, and are therefore indispensable for numerous mitochondrial bioenergetic processes [8,9,10,11]. In addition, CLs ensure mitochondrial quality control through the regulation of mitochondrial autophagy (mitophagy) [12]. Moreover, because of the presence of unsaturated fatty acyl chains, CLs are sensitive to oxidative stress. Oxidation of its fatty acyl chain could provide cell death signals and control mitochondrial apoptosis [13]. In fact, the balance between the saturated and unsaturated fatty acyl chain is critical for regulating cellular apoptosis, in particular, in response to therapeutic drugs. Dysregulation of CL metabolism has been observed in several types of cancer and, thus, proposed as a potential therapeutic target. In this review, we describe the main mitochondrial functions in which CLs play a pivotal role. We also discuss different types of cancer in which CLs or CL metabolism enzymes are found to be dysregulated and, thus, could be involved in the development of the disease.

## 2. Cardiolipin Metabolism

**Figure 2:**
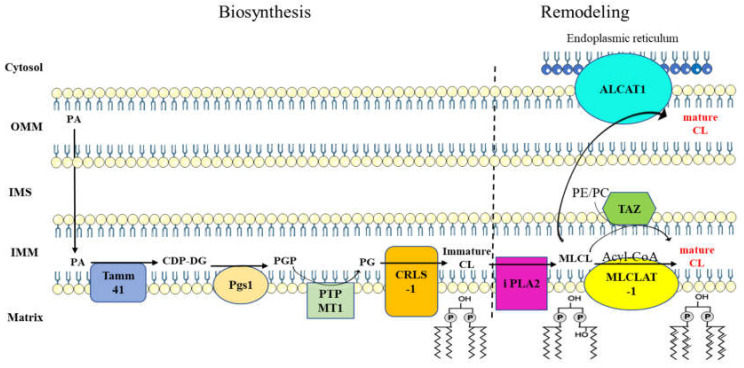
Cardiolipin metabolism is a two-step process. Biosynthesis step results in the formation of immature CL with acyl chain composition that is not yet definitive. Fatty acyl chain of immature CL undergoes several modifications through the activity of remodeling proteins, leading to the formation of mature CL. Abbreviations: PA, phosphatidic acid; Tamm41 (CDP-DS), CDP diacylglycerol synthase; CDP-DG, cytidine diphosphate-diacylglycerol; Pgs1, phosphatidylglycerophosphate synthase 1; PGP, phosphatidylglycerol phosphate; PTPMT1 (PGPP), phosphatidylglycerophosphate phosphatase 1; PG, phosphatidylglycerol; CRLS1, cardiolipin synthase 1; CL, cardiolipin; PLA2, phospholipase A2; MLCL, monolysocardiolipin; MLCLAT1, monolysocardiolipin acyltransferase 1; TAZ, tafazzin; ALCAT1, acyl-CoA:lysocardiolipin acyltransferase; OMM, outer mitochondrial membrane; IMM, inner mitochondrial membrane; IMS, intermembrane space; PE, phosphatidylethanolamine; PC, phosphatidylcholine.

CL metabolism is divided into two main steps (Figure 2). The first step consists of its biosynthesis. It is initiated upon the formation of phosphatidic acid (PA), which takes place in the outer mitochondrial membrane (OMM) and is then transferred into the inner mitochondrial membrane (IMM). Once in the IMM, the conversion of PA into CDP-diacylglycerol (CDP-DG) is effected by a CDP-DG synthase protein also known as Tamm41 protein [14]. CDP-DG is then converted to phosphatidylglycerol phosphate (PGP) by the phosphatidyl glycerophosphate synthase 1 (Pgs1) enzyme which catalyzes the transfer of a phosphatidyl group to the glycerol-3-phosphate (G3P) [15]. This pathway is then pursued by the phosphatidyl glycerophosphate phosphatase 1 (PGPP1) enzyme to form phosphatidylglycerol (PG) [16]. Finally, CL synthase 1 (CRLS1) catalyzes the formation of immature CL by using CDP-DG and PG [17]. Immature CLs are characterized by saturated acyl chains of variable length and asymmetry around the glycerol head group [18,19]. CL biosynthesis is then followed by structural modifications called remodeling. This step starts by removing an acyl chain via the activity of a phospholipase to form monolyso-CL (MLCL). The MLCL is then reacylated by an acyl transferase to form mature CL. Three enzymatic pathways are involved in CL remodeling. Tafazzin is a transacylase localized on the outer face of the IMM and transfers an acyl chain of phosphatidylethanolamine (PE) and/or phosphatidylcholine (PC) to MLCL, allowing the formation of a fully mature form of CL [20,21]. The acyl-CoA:lysocardiolipin acyltransferase (ALCAT1) and MLCL acyltransferase 1 (MLCLAT1) are two other acyltransferases that reside, respectively, on the endoplasmic reticulum mitochondria-associated membranes (ER MAM) and inner leaflet of the IMM. Both ALCAT1 and MLCLAT1 use the acyl chain of acyl-CoAs to ensure MLCL reacylation [22,23]. Moreover, studies show the involvement of mitochondrial protein complexes and supercomplexes in the remodeling process [24,25]. Among the mitochondrial protein complexes, three main groups are known to be involved: Prohibitins [26], the mitochondrial contact site, and the cristae organizing system (MICOS) complex [27,28] and the OXPHOS complexes [29,30,31]. Mature CLs that are characterized by symmetric incorporation of unsaturated longer fatty acyl chains are then assembled into mitochondrial protein complexes, allowing supercomplexes’ formation and assembly [19,32]. This interaction with mitochondrial protein complexes and supercomplexes protects CL from degradation and confers a long half-life to CL [33].

Dysregulation in biosynthesis and remodeling of CL impair proper mitochondrial function. Therefore, abnormalities in CL metabolism can be associated with pathophysiological conditions including cancer. Several studies showed changes in CL content and/or composition in tumor tissues or cancer cells. In human prostate cancer tissues, changes in the quantity of various lipids including CL were observed, and CL content was found to be increased in the regions with high tumor cell density [34]. In addition, a modification of CL composition has been observed in tumor prostate tissue of patients [34]. Thus, in tumor tissue, CL composition in palmitoleic acid (C16:1) was increased compared with non-tumor tissue. Increasing the CL content in palmitoleic acid was positively correlated with the proliferation of PC-3 cells. Furthermore, a diet rich in oleic acid (C18:1) decreases PC-3 cell proliferation by changing the fatty acid composition of CL [35]. High CL quantity and alteration of acyl chain composition are also observed in thyroid oncotic tumors and are considered to be a tumor biomarker [36]. However, a direct correlation between higher CL content and cancer aggressiveness does not seem to be true in all types of cancer. For instance, in human tumor tissues of hepatocellular carcinoma (HCC), CL content was found to be gradually decreased during HCC progression compared to peripheral non-cancerous tissues, accompanied by a concomitant decrease of oxidized CL [13]. Additionally, CL species of tetralinoleoyl CL (TLCL) decrease in three liver cancer cell lines (Huh7, HepG2, LM3) while the level of saturated and monounsaturated CL is increased compared to non-cancer cells [13]. All those observations give rise to the idea that CL acyl chains are more important than CL content and could play a key role in the control of cancer aggressiveness. In fact, a decrease in the polyunsaturated fatty acyl chain can decrease CL fatty acyl oxidation, which may disrupt apoptotic response. However, how CL fatty acyl chains control tumor cell proliferation remains unclear.

The study of CL metabolism proposes a tumor suppressor implication of the CL biosynthesis gene, CRLS1. Thus, in patients with non-small cell lung cancer (NSCLC), a positive correlation between high CRLS1 mRNA expression and their overall survival was described [37]. CRLS1 was co-expressed with PPM1A and PTPRR, genes that are involved in the mitogen-activated protein kinase (MAPK) signaling pathway [37]. PPM1A is a phosphatase involved in tumor suppression via dephosphorylation of MAPKs, P38, and JNK [38,39]. Similarly, the tumor suppressor function of PTPRR in a cervical cancer model has been suggested [40]. More studies also propose the tumor suppressor activity of CRLS1 in HCC. It has been found that LINC00961, a novel long non-coding RNA which has been uncovered as a tumor suppressor in lung cancer and glioma, was downregulated in HCC tissues and cell lines (HepG2 and Hep3B) [41]. Mechanistically, in HepG2 and Hep3B HCC cell lines, overexpression of long non-coding RNA LINC00961 was proportionally correlated with CRLS1 mRNA and protein overexpression, resulting in the inhibition of HCC progression (i.e., inhibition of cell proliferation, migration, and invasion in HCC cells). Both LINC00961 and CRLS1 protein expression were downregulated in patients’ HCC tumors [41]. These studies suggest a tumor suppressor function for CRLS1 that is co-expressed with other tumor suppressor proteins. However, how this CL metabolism enzyme could impact CL metabolism along with its tumor suppressor function and the downstream pathways leading to the inhibition of tumor growth is not clearly understood. CRLS1 is not the only CL metabolism enzyme that could be involved in cancer. Contrary to CRLS1, studies propose a tumorigenesis function for CL remodeling enzyme, tafazzin. The level of tafazzin protein expression was found to be increased in squamous cervical carcinoma compared to normal cervical tissue [42]. In cervical cancer cells, overexpression of tafazzin significantly increased cell growth and viability. Moreover, tafazzin silencing increased apoptosis in cervical cancer cells, suggesting an inhibitor role on apoptosis for tafazzin [42]. It seems that tafazzin controls mitochondrial apoptotic pathways as its knockdown increases the cleavage of caspase 9 and caspase 3, the two proteins involved in the intrinsic apoptotic pathway. In addition, the tumorigenesis role of tafazzin has been proposed in rectal cancer and thyroid cancer where tafazzin expression was related to cancer development and its suppression has led to thyroid cancer apoptosis [43,44].

As a phospholipid signature of mitochondria, CL has a key role in the stability and integrity of mitochondrial structure and function. There is growing evidence that reprogramming of CL metabolism exists in cancer. However, whether and how CL (via resistance to anti-tumor drugs, tumor growth, and metastasis) really influences cancer needs to be determined. Indeed, in most cases, the evidence for a role for CL in cancer is merely correlative, suggestive, ambiguous, and cancer-type dependent. In addition, although some enzymes of CL biosynthesis and remodeling were found to be involved in the control of cancer inhibition/development, no information on CL content and composition was found. Therefore, it seems that CL diversity and the expression levels of individual CL metabolism enzymes are not significantly linked.

## 3. Role of CL in Metabolic Reprogramming

In mitochondria, energy is produced through the OXPHOS process [45]. The four complexes (I–IV) of the respiratory chain catalyze the electron transport from reduced equivalents (nicotinamide adenine dinucleotide (NADH) and flavin adenine dinucleotide (FADH2)) to the oxygen molecule and create a transmembrane proton gradient. This gradient provides an energy source to produce ATP by complex V (ATP synthase). Metabolic reprogramming in most of the cases supports tumor growth, favors cancer invasion and metastasis and response to therapy, and is now recognized as one of the hallmarks of cancer [46]. It is now clear that this metabolic plasticity requires fully functional mitochondria and finely-tuned regulations of their activity.

CL interacts with protein complexes of the ETC and OXPHOS and enables their structural integrity and proper enzymatic activity. Mitochondria with impaired CL biosynthesis have an altered cristae structure and the assembly of the ETC supercomplex is decreased [10,11]. The presence of CL-specific binding sites has been detected in complexes I, III, and IV [10,47]. ETC protein complexes are organized into the higher organized structures known as supercomplexes or « respirasome » in order to improve the electron transfer efficiency [48,49]. The association of individual complexes into supercomplexes requires the interaction with CL to be established [50,51]. The effect of CL remodeling and quantity on mitochondrial bioenergetics has been proposed in cancer-related cachexia, which is driven by a reduced food intake with increased energy expenditure, excess catabolism, and inflammation, and leads to treatment-related complication, poor quality of life, and cancer-related mortality [52]. In urothelial-carcinoma-related cachexia, mitochondrial phospholipid remodeling including CL was associated with lower respiratory chain activity and higher expression of mitochondrial UCP3 in skeletal muscle, resulting in a decrease in ATP production [53]. Moreover, in a rat model of cancer cachexia induced by peritoneal carcinosis, higher CL content and acyl chain modification has been observed in liver mitochondria and was accompanied by lower efficiency of oxidative phosphorylation and an increase in energy wasting [54]. Functionally, enrichment of CL content in the Hepa-RG hepatocyte-like cells leads to an increase in mitochondrial oxygen consumption with higher energy wasting [55]. Whether CL can play a direct role in cancer cachexia by modulating metabolism needs to be studied further.

On the other hand, a decrease in CL content and modification of CL molecular species has been observed in the mitochondria from CT-2A (malignant anaplastic astrocytoma) and EPEN (ependymoblastoma) brain tumors compared with the mitochondria isolated from B6 mouse brain [56]. Moreover, in the VM-NM1 (a rapidly growing nonmetastatic tumor) and VM-M2 (highly invasive tumor) brain tumors, CL content was lower than in the control VM mouse brain, and its acyl chain composition was modified in all VM-NM1, VM-NM2, and VM-NM3 (invasive tumor) brain tumors. In all mentioned mouse brain tumor types, the activity of mitochondrial respiratory chain complexes I and II, as well as overall activity of mitochondrial ETC, was decreased [56]. In addition, in C6 glioma cells, tafazzin knock-down in the presence of linoleic acid reduced the capacity of oxidative phosphorylation [57], mitochondrial complex I function, and increased glycolytic activity [58]. All those changes were accompanied by a dramatic reduction in cell proliferation [57,58]. It has already been demonstrated that the decrease in CL content affects the assembly of the ETC supercomplex [10]. Moreover, complex I assembly into supercomplexes can regulate mitochondrial reactive oxygen species (ROS) production [59]. Therefore, it could be hypothesized that the alteration in the ETC supercomplex assembly increases the production of ROS, which will regulate subsequent signaling pathways such as cell proliferation and apoptosis.

CL also seems required for acetyl CoA synthesis and is essential for the normal function of the tricarboxylic acid (TCA) cycle [60,61]. In this line, the CL-deficient model of a mouse C2C12 myoblast cell line has shown a decreased carbon flux from glucose to acetyl CoA associated with a decrease in pyruvate dehydrogenase activity [60]. Moreover, the implication of acetyl CoA in cancer cell migration and metastasis has already been demonstrated [62,63,64,65,66]. For instance, studies show that high levels of acetyl CoA could increase the acetylation and activation of the Smad2 transcription factor, resulting in epithelial-mesenchymal transition and induction of metastasis in breast cancer cell lines [62]. Additionally, in pancreatic ductal adenocarcinoma, increases in the acetyl CoA level favor cell proliferation through histone acetylation [65,66]. These studies suggest a link between CL, TCA cycle function, and energy metabolism, which consequently influence tumor cell proliferation and metastasis.

Because of its crucial role in the assembly of the mitochondrial protein complexes and supercomplexes, it is conceivable that any alteration in CL metabolism could influence respiratory chain activity and efficiency of oxidative phosphorylation and in turn metabolic reprogramming in cancer or cancer cachexia. However, more studies are required to reveal the fundamental mechanism through which CL, by modulating metabolism, may influence cancer aggressiveness (i.e., migration/invasion and resistance to treatment). In addition, mitochondrial fatty acid oxidation is another strategy used by cancer cells to promote proliferation, drug resistance, and metastatic progression [67]. Interestingly, both fatty acid oxidation and CL remodeling required a monolysocardiolipin acyltransferase-like enzyme, hydroxyacyl-CoA dehydrogenase trifunctional multienzyme complex subunit alpha (HADHA) (tri-functional protein alpha), to ensure proper mitochondria function in human cardiomyocytes [68]. However, their direct interplay in cancer is not fully elucidated.

## 4. Role of CL in Mitochondrial Quality Control

The proper function of mitochondria requires the maintaining of their proper architecture that is ensured by the mitochondrial quality control mechanism. This mechanism is under the control of two main processes, mitochondrial dynamics and mitophagy. Mitochondrial dynamics regulate the quality control mechanism through mitochondrial fusion and fission processes [69]. Mitochondria fusion allows two mitochondria to merge into one single mitochondrion. This process is initiated by the activation of dynamin-related GTPases, mitofusin (Mfn-1 and Mfn-2) and optic atrophy (OPA-1), allowing the fusion of OMM and IMM, respectively [70,71]. Even if the exact underlying mechanisms are still being studied, it has been proposed that mitochondrial fusion could play a role in resistance to therapy. In fact, increased mitochondrial fusion was found to be associated with cisplatin-resistance of cervical and ovarian cancer cell lines [72]. This finding suggests that fused mitochondria may have better ETC and OXPHOS protein activity and, thus, a better ATP production which may be responsible for cell survival and resistance. During fission, one mitochondrion undergoes the division into daughter mitochondria via the activity of dynamin-related protein 1 (DRP1) [71]. DRP1 recruitment to the OMM leads to the formation of a ring-like structure around mitochondria followed by mitochondria division [73]. Although mitochondrial fission is required to ensure mitochondrial dynamics, it may intensify cancer aggressiveness. For example, it has been found that invasive breast carcinoma cell lines have fragmented mitochondria and a higher DRP1 level than a non-metastatic cell line. Moreover, DRP1 silencing was associated with decreased migration and invasion [74]. In addition, in a glioblastoma cell line, the expression of DRP1 and mitochondria fission was increased in the hypoxic condition associated with higher migration and invasion. Treatment with DRP1 inhibitor (M-DIVI-1) decreased hypoxia-induced migration [75]. CL is critical for the mitochondrial fusion process by ensuring the OPA-1 biogenesis and assembly [76]. Interaction of CL with Opa1 stimulates GTPase activity of this protein, ensuring mitochondrial fusion [77,78,79]. Studies suggest that mitochondrial fusion can be influenced by CL content and composition. An in vitro membrane fusion reaction has been performed, showing that the incubation of CL-containing liposomes with purified human OPA-1 protein promotes membrane fusion, while no effect was observed with low concentrations of CL. Furthermore, the increase in the unsaturated acyl chain of CL could improve this process [76]. CL interaction with DRP1 seems to be crucial in promoting mitochondrial fission [80,81]. In fact, it has been shown that DRP1 interacts preferentially with membranes containing CL compared to membranes containing other anionic lipids. Moreover, the interaction of DRP1 with CL has been found to be essential to stimulate its GTPase activity [80].

**Figure 3:**
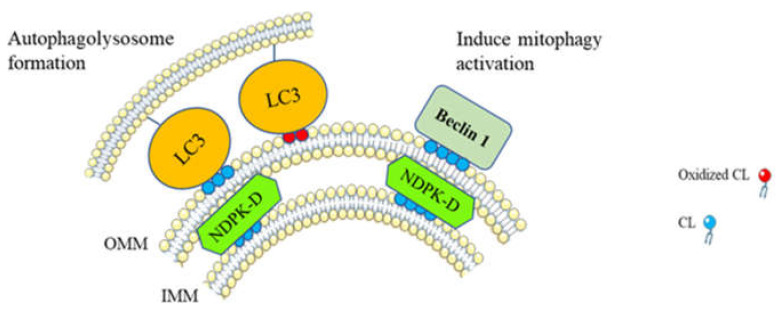
Cardiolipin translocation to the outer mitochondrial membrane (OMM) induces mitophagy. In damaged mitochondria, translocation of CL to the OMM is performed via nucleoside diphosphate kinase D (NDPK-D) activity [94]. CL on the OMM interacts with the cytosolic mitophagic protein LC3 which is responsible for autophagolysosome formation [12] and beclin 1, leading to the activation of other mitophagic actors [98]. Moreover, oxidized CL can be dissociated from cytochrome c and therefore translocated to the OMM, where their interaction with LC3 initiates the formation of autophagolysosome [95].

In the damaged mitochondrion, the impaired daughter mitochondria produced by the fission mechanism can be further eliminated by mitochondrial-specific autophagy known as mitophagy. Mitophagy is a process by which damaged and dysfunctional mitochondria are selectively degraded via autophagy [82]. In stress conditions such as mitochondrial membrane depolarization, hypoxia, or nutrient deprivation, activation of mitophagy maintains mitochondrial integrity and function [83]. Inversely, defective mitophagy can lead to the development of diseases including cancer. Indeed, tumor suppressor activity of mitophagy has already been demonstrated in numerous types of cancers [84,85,86,87,88,89]. However, depending on the stages of tumor development, mitophagy may also be involved in tumorigenesis by promoting tumor cell survival and metastasis [90,91,92,93]. CL is known to be involved in the modulation of mitophagy in order to regulate the mitochondrial quality control process (Figure 3). In the damaged mitochondria, CL migrates from the IMM to the OMM via nucleoside diphosphate kinase D (NDPK-D) [12,94]. Externalized CLs specifically interact with light chain 3 (LC3) protein which allows the recognition of injured mitochondria and initiates the formation of autophagolysosome [12]. Moreover, CL peroxidation allows for the translocation of oxidized CL to the OMM. Oxidized CL on the OMM can be recognized by a LC3 subfamily, LC3A, which may prevent CL recognition by apoptotic machinery [95]. Downregulation of CL biosynthesis, preventing CL localization at the OMM, and blocking the LC3–CL interaction, inhibits LC3-dependent mitophagy [96]. In addition, modification of CL composition, mainly a decrease in TLCL, results in the accumulation of dysfunctional and damaged mitochondria [12,97]. CL on the OMM can directly interact with beclin 1 in order to induce mitophagy [98]. The role played by mitophagy via CL in cancer is understudied. In chronic myeloid leukemia (CML), CL interacts with the PH-domain of p210 BCR-ABL protein (a common variant causing CML). This interaction allows for the translocation of p210 BCR-ABL protein from the cytosol to the mitochondria and promotes mitochondrial damage. Damaged mitochondria can further be eliminated via mitophagy [99].

It seems important to maintain a balance of fusion and fission as mitochondrial fusion could be associated with therapy resistance of cancer cells [72], and mitochondrial fission seems to be rather involved in cancer invasion [74,75]. Interaction of CL with fusion and fission mitochondrial proteins has been studied. However, little is known about the implication of CLs in fusion and fission mechanisms that are related to the resistance and invasion in cancer cells. Similarly, despite its implication in the activation of mitophagy, there are not enough data about whether the interaction of CLs with mitophagy actors could be implicated in cancer disease and how CL-dependent mitophagy is modified at different stages of cancer development. Therefore, to clearly understand how CL could be involved in mitochondrial dynamics/mitophagy related abnormalities, more investigations are required.

## 5. Role of CL in Apoptosis

**Figure 4:**
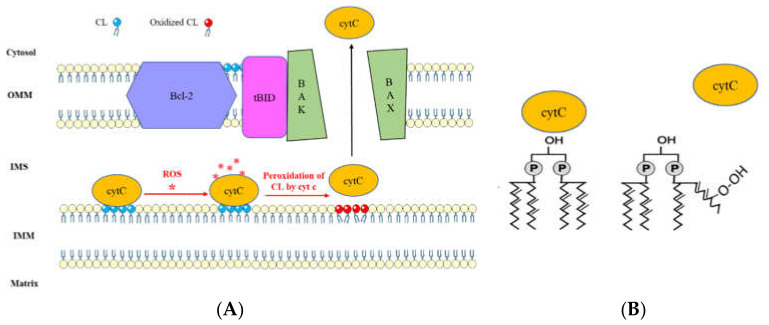
(**A**) Cardiolipin-mediated apoptotic pathway via Bcl-2 family proteins interaction and cytochrome c release. Production of reactive oxygen species (ROS) under stress signals confer a peroxidase activity to cytochrome c (cytC). Oxidation of CL via cytC results in dissociation of cytC-CL complex, leading to cytC release to the cytosol [116,117]. Moreover, the interaction of CL with tBID allows the oligomerization of Bax/Bak, resulting in mitochondria disruption and cytC release to the cytosol, which will activate the caspase signaling pathway and initiate apoptosis [104,105,106]. (**B**) Schematic representation of CL peroxidation which leads to dissociation of CL-cytC complex.

Mitochondria play a pivotal role in the accomplishment of apoptotic signals [100,101]. Permeabilization of the OMM leads to the release of apoptotic factors from the mitochondria to the cytosol and induces apoptosis [100,102,103]. It has been found that permeabilization of mitochondrial OMM is tightly regulated by the proteins from the Bcl-2 family. A possible CL-dependent apoptotic pathway is mediated through caspase-8/tBID cooperation [104]. During this pathway, activation of caspase-8 leads to cleavage of BH3 interacting domain death agonist (BID), producing an active carboxyl fragment (tBID) which is translocated to the mitochondria and, by cooperation with BAX protein, promotes permeabilization of mitochondrial membrane and cytochrome C (cytC) release [104,105,106]. It has been suggested that CL provides an activating platform for caspase-8 translocation on mitochondria and thereby seems necessary for integral activation of caspase-8 and efficient apoptotic response [107,108]. Moreover, studies suggested that CLs are involved in the transduction of apoptotic signals by interacting with tBID protein [109] (Figure 4). Interaction of CL with tBID leads to the modification of the mitochondrial structure. This modification allows for the oligomerization of Bax/Bak through the BH3 domain of tBID, resulting in the disruption of the mitochondrion [110]. Moreover, tBID/CL interaction by remodeling the cristae structure can promote the cytC release to the cytosol which in turn activates caspase 9 and initiates apoptosis [111,112].

Through their electrostatic interaction with cytC, CLs ensure electron transfer in the ETC [113]. This interaction seems thus indispensable for the OXPHOS process [114]. In addition, the unsaturated acyl chain of CL can interact through a hydrophobic interaction with cytC [113]. This hydrophobic interaction between CL/cytC confers a peroxidase activity to cytC, resulting in the oxidation of the CL acyl chain [115]. During apoptosis, oxidized CL has a very low affinity with cytC, leading to detachment of cytC from the complex and its release to the cytosol [116,117]. The impact of CL-cytC complexes in apoptosis has been studied in ovarian cancer cells. In the doxorubicin-sensitive and -resistant human ovarian cancer cells (A2780 and A2780-ADR, respectively), treatment with cytC-CL nano-complexes have induced apoptosis while a free form of cytC was not sufficient to promote cell death. CL-cytC complex-induced cell death was due to a lipoperoxidase reaction achieved by CL-coupled cytC [118]. In HCC, CL was significantly decreased in human tumor tissues compared with the non-tumor area, accompanied by a decrease in oxidized CL level. CL profiles of Huh7 and HepG2 HCC cell lines revealed a decrease in the TLCL CL species level with an increase in saturated and monounsaturated CL compared to non-cancer cells. The increase in CL content with a high level of TLCL species intensifies the production of CL oxidation products increasing apoptotic sensitivity in response to the sorafenib [13]. More studies on Huh7 cell lines have shown that under lysosomal stress, CL content was decreased, leading to mitochondrial fission. Mitochondrial fission was accompanied by an impaired mitochondrial function associated with mitochondria-induced apoptosis [119]. CLs also have a high affinity with the anticancer drug doxorubicin (Dox). Thereby, the CL–Dox interaction can allow for the internalization of Dox into mitochondria, leading to cell toxicity and death. In this line, studies on Barth syndrome (X-linked recessive disease caused by a mutation in the tafazzin gene) showed that the B-lymphocytes of Barth syndrome patients are less sensitive to Dox-induced oxidative stress, while CL content is decreased [120].

Due to its fatty acid composition, CL is subject to chemical modification and oxidation. Moreover, its specific interaction with cytochrome C and likely with some Bcl-2 family members makes CL pivotal in regulating mitochondrial apoptosis. Modification of CL content and acyl chain composition may directly impact apoptosis in cancer cells [13]. It can be hypothesized that lower CL content, by decreasing OMM permeabilization, inhibits apoptosis signal transmission. Additionally, a decrease in unsaturated fatty acyl content by reducing CL peroxidation could reduce apoptosis [13]. However, the real role played by CL-related apoptosis and the underlying mechanism by which cancer cells modify their CL content/composition remains unclear and requires more revealing studies.

## 6. Conclusions

CL could play a pivotal role in several mitochondrial functions/parameters such as bioenergetics, dynamics, mitophagy, and apoptosis, which are involved in key steps of cancer aggressiveness (i.e., migration/invasion and resistance to treatment). In addition, several studies suggest that CL metabolism reprogramming occurs in cancer cells. A better understanding of why the CL profile is different in cancer cells and how CL controls tumor metastasis and drug resistance may not only be instrumental for the clinical relevance of studying CL metabolism, but also for the development of innovative therapeutic approaches for cancer.

## Abbreviations

ADR	Adriamycin
ALCAT1	Acyl-CoA:lysocardiolipin acyltransferase
ATP	Adenosine triphosphate
BAK	Bcl-2 antagonist or killer
BAX	Bcl-2 associated X protein
Bcl-2	B-cell lymphoma-2
BID	BH3 interacting domain death agonist
CDP-DG	Cytidine diphosphate diacylglycerol
CL	Cardiolipin
CML	Chronic myeloid leukemia
CRLS1	Cardiolipin synthase 1
CytC	Cytochrome C
DOX	Doxorubicin
DRP1	Dynamin-related protein 1
ETC	Electron transfer chain
ER	Endoplasmic reticulum
FADH2	Flavin adenine dinucleotide (reduced)
G3P	Glycerol-3 phosphate
HADHA	Hydroxyacyl-CoA dehydrogenase trifunctional multienzyme complex subunit alpha
HCC	Hepatocellular carcinoma
IMM	Inner mitochondria membrane
JNK	c-Jun N-terminal kinase
LC3	Light chain 3 protein
MAM	Mitochondria-associated membrane
MAPK	Mitogen-activated protein kinases
Mfn	Mitofusin protein
MICOS	Mitochondrial contact site and cristae organizing system
MLCL	Monolyso-cardiolipin
MLCLAT1	Monolyso-cardiolipin acyl transferase 1
NADH	Nicotinamide adenine dinucleotide (reduced)
NDPK-D	Nucleoside diphosphate kinase D
NSCLC	Non-small cell lung cancer
OMM	Outer mitochondria membrane
OPA-1	Optic atrophy-1
OXPHOS	Oxidative phosphorylation system
P38	P38 Mitogen-activated protein kinases signaling pathway
PA	Phosphatidic acid
PC	Phosphatidyl choline
PE	Phosphatidyl ethanolamine
PG	Phosphatidyl glycerol
PGP	Phosphatidyl glycerol phosphate
PGPP1	Phosphatidyl glycerophosphate phosphatase 1
Pgs1	Phosphatidyl glycerophosphate synthase 1
PPM1A	Protein phosphatase, Mg2+/Mn2+ dependent 1A
PTPRR	Protein tyrosine phosphatase receptor type R
TCA cycle	Tricarboxylic acid cycle
UCP3	Mitochondrial uncoupling protein 3
